# Quorum Sensing Signaling and Quenching in the Multidrug-Resistant Pathogen *Stenotrophomonas maltophilia*

**DOI:** 10.3389/fcimb.2018.00122

**Published:** 2018-04-24

**Authors:** Pol Huedo, Xavier Coves, Xavier Daura, Isidre Gibert, Daniel Yero

**Affiliations:** ^1^Institut de Biotecnologia i de Biomedicina, Universitat Autònoma de Barcelona, Barcelona, Spain; ^2^Departament de Genètica i de Microbiologia, Universitat Autònoma de Barcelona, Barcelona, Spain; ^3^Catalan Institution for Research and Advanced Studies, Barcelona, Spain

**Keywords:** multi-drug resistance, quorum sensing, quorum quenching, nosocomial infections, antimicrobial resistance

## Abstract

*Stenotrophomonas maltophilia* is an opportunistic Gram-negative pathogen with increasing incidence in clinical settings. The most critical aspect of *S. maltophilia* is its frequent resistance to a majority of the antibiotics of clinical use. Quorum Sensing (QS) systems coordinate bacterial populations and act as major regulatory mechanisms of pathogenesis in both pure cultures and poly-microbial communities. Disruption of QS systems, a phenomenon known as Quorum Quenching (QQ), represents a new promising paradigm for the design of novel antimicrobial strategies. In this context, we review the main advances in the field of QS in *S. maltophilia* by paying special attention to Diffusible Signal Factor (DSF) signaling, Acyl Homoserine Lactone (AHL) responses and the controversial Ax21 system. Advances in the DSF system include regulatory aspects of DSF synthesis and perception by both *rpf*-1 and *rpf*-2 variant systems, as well as their reciprocal communication. Interaction via DSF of *S. maltophilia* with unrelated organisms including bacteria, yeast and plants is also considered. Finally, an overview of the different QQ mechanisms involving *S. maltophilia* as quencher and as object of quenching is presented, revealing the potential of this species for use in QQ applications. This review provides a comprehensive snapshot of the interconnected QS network that *S. maltophilia* uses to sense and respond to its surrounding biotic or abiotic environment. Understanding such cooperative and competitive communication mechanisms is essential for the design of effective anti QS strategies.

## Introduction

*Stenotrophomonas maltophilia* is a ubiquitous multidrug resistant Gram-negative bacterium that has emerged as an important nosocomial pathogen (Brooke, 2012; Adegoke et al., 2017) and stands as one of the most common lung pathogens in cystic fibrosis patients (Amin and Waters, 2014). The most important natural reservoir of this microorganism is thought to be the rhizosphere (Berg et al., 2005; Ryan et al., 2009), a highly competitive niche that facilitates the acquisition by bacteria of antimicrobial-resistance genes (Berg et al., 2005) and favors the establishment of communication networks between neighboring organisms (Bais et al., 2006). The result of this competitive coevolution appears to have a strong impact when translated to clinical environments.

Bacterial cells can communicate through the production and detection of signal molecules, a mechanism known as quorum sensing (QS) (Waters and Bassler, 2005; Papenfort and Bassler, 2016). Through cell-to-cell communication, bacterial populations synchronize gene expression and globally respond to changes in the environment, also during infection (Rutherford and Bassler, 2012). QS communication may also connect different bacterial species and even members of different kingdoms (Lowery et al., 2008). On the other end, the disruption of exogenous QS, a phenomenon termed Quorum Quenching (QQ), constitutes a varied and widespread protection mechanism exploited by bacterial competitors and by host defenses in case of infection (Dong et al., 2007). Indeed, interrupting bacterial QS strongly impairs bacterial pathogenic capacity (Kalia and Purohit, 2011).

Several different QS signals and QQ mechanisms have been identified in the last decades, significantly expanding our knowledge on bacterial communication (Kleerebezem et al., 1997; Dong et al., 2007; Deng et al., 2011; Kalia and Purohit, 2011; Ryan et al., 2015; Papenfort and Bassler, 2016; Zhou et al., 2017). Here, we review recent advances in the characterisation of the QS network of *S. maltophilia*, focusing on the two variants regulating the diffusible signal factor (DSF) system, as well as the QQ mechanisms in which this microorganism is involved. We also discuss the role of N-acyl homoserine lactone (AHL) signaling molecules and the controversial Ax21 system in the QS network of this species. Overall, this review provides a comprehensive picture of the signaling network that interconnects *S. maltophilia* with its surrounding environment.

## DSF-quorum sensing in *S. maltophilia*

So far, the most studied QS system in *S. maltophilia* is that based on the DSF fatty acid (FA) signal *cis*-11-methyl-2-dodecenoic acid, originally described in *Xanthomonas campestris* pv. *campestris* (*Xcc*) (Barber et al., 1997). As a *Xanthomonadales* member and differently than the unrelated DSF-like-producing bacteria *Burkholderia cenocepacia and Pseudomonas aeruginosa, S. maltophilia* governs DSF communication through the genes co-localized in the *rpf* (regulation of pathogenicity factors) cluster (Huedo et al., 2015). Genes within this cluster include key enzymes of DSF synthesis, perception and signal transduction and are organized in two adjacent operons that are convergently transcribed. One operon is composed by the genes encoding for the FA ligase RpfB and the synthase RpfF, while the opposite operon encodes for a two-component system including the sensor kinase RpfC and the cytoplasmic regulator RpfG (Fouhy et al., 2007; Huedo et al., 2014b). Unlike *Xanthomonas* sp. and similar to *Xylella fastidiosa*, the *rpf* cluster in *S. maltophilia* does not encode for the transmembrane protein RpfH (Huedo et al., 2014b).

### Two *rpf* cluster variants in *S. maltophilia*

A distinctive feature of the DSF system in *S. maltophilia* is the presence of two *rpf* cluster variants that produce and sense DSF signals distinctly and regulate important biological processes (Huedo et al., 2014b). Two initial studies investigating the relation between genotypic and phenotypic traits of *S. maltophilia* isolates suggested that a significant group of strains lacked the *rpfF* gene (Pompilio et al., 2011; Zhuo et al., 2014). Later, a population study focused on DSF-QS revealed that, unlike the other *Xanthomonadales, S. maltophilia* presents two *rpfF* variants (named *rpfF*-1 and *rpfF*-2) and that primers designed to PCR-amplify the *rpfF* gene didn't recognize the *rpfF*-2 variant (Huedo et al., 2014b). More recently, the existence of the two *rpfF* alleles in *S. maltophilia* clinical and environmental isolates has been further validated by a population genomic analysis (Lira et al., 2017). The two *rpfF* variants differ, in particular, in the sequence encoding for the N-terminal 108 residues (Huedo et al., 2014b). Taking all the published data together (Huedo et al., 2014b; Lira et al., 2017) and assuming that the *rpfF*^−^ isolates from Pompilio et al. (2011) and Zhuo et al. (2014) belong to the *rpfF*-2 variant, the *rpfF*-1 variant has been so far identified in 98 isolates (55.5%), while *rpfF*-2 has been detected in 81 isolates (44.5%).

Investigation of the *rpf* cluster in the two *rpfF* variant strains showed that the sensor RpfC presents two variants as well, with a fixed association between the *rpfF* variant and its cognate *rpfC*, meaning that all strains harboring *rpfF*-1 necessarily carry the *rpfC*-1 variant and likewise for the *rpfFC*-2 pair (Huedo et al., 2014b). Besides differences in amino-acid sequence, the two RpfC variants vary in length and secondary structure. RpfC-1 displays 10 trans-membrane regions (TMR) in the N-terminal region that are highly related to the RpfC-RpfH complex constituting the DSF sensor domain in *Xcc* (Slater et al., 2000; Huedo et al., 2014b). On the contrary, RpfC-2 lacks 5 of these TMRs as in *Xylella fastidiosa* (*Xf*) RpfC (Chatterjee et al., 2008; Huedo et al., 2014b). Differences between the *rpf* cluster variants strongly affect DSF synthesis, perception, and regulation of biological processes in *S. maltophilia*.

### *rpf*-1 and *rpf*-2 strains distinctly synthesize and sense DSF signals

Remarkably, while *rpf*-1 strains display evident DSF production in standard growth conditions, *rpf*-2 isolates require extra copies of *rpfF-*2 or the absence of the sensor/repressor component RpfC-2 to achieve detectable levels of DSF (Huedo et al., 2014b). The mechanistic aspects of DSF synthesis and perception in *S. maltophilia rpf*-1 seem to be similar to those reported for the model organism *Xcc*. Both microorganisms synthesize *cis*-11-methyl-2-dodecenoic acid as the main DSF signal (He and Zhang, 2008; Huedo et al., 2014b). *Xcc* RpfF produces additional DSF signals including *cis*-2-dodecenoic acid, *cis*-11-methyldodeca-2,5-dienoic acid, and *cis*-10-methyl-2-dodecenoic acid (Deng et al., 2015, 2016; Zhou et al., 2015). The production of seven derivatives of the *cis*-11-methyl-2-dodecenoic acid by one *S. maltophilia* strain (WR-C) had been also reported (Huang and Lee Wong, 2007). More recently, however, the canonical *cis*-11-methyl-2-dodecenoic acid was the only unsaturated FA signal identified in culture supernatants of the *S. maltophilia* strains E77 (RpfF-1) and M30 (RpfF-2) (Huedo et al., 2014a,b) (Table 1).

**Table 1:** *Stenotrophomonas maltophilia* strains in which the diffusible signal factor (DSF) quorum sensing (QS) system has been investigated.

**Strain**	**Origin**	**RpfF variant**	**DSF molecules**	**Biological processes**	**References**
K279a	Clinical (blood infection)	1	*cis*-11-Methyl-2-dodecenoic acid (DSF)	Motility; Protease production; Lipopolysaccharide synthesis; Antimicrobial resistance; OMV production; Virulence	Fouhy et al., 2007; Devos et al., 2015
WR-C	Environmental (septic tank)	NA*	*cis*-11-Methyl-2-dodecenoic acid (DSF); Δ2-tridecenoic acid; 11-methyl-dodecanoic acid; 10-methyl-dodeccanoic acid; Δ2-12-methyl-tridecenoic acid; Δ2-tetradecenoic acid; Δ2-12-methyl-tetradecenoic acid; Δ2-13-methyl-tetradecenoic acid	Motility	Huang and Lee Wong, 2007
E77	Clinical (sputum)	1	*cis*-11-Methyl-2-dodecenoic acid (DSF)	Motility; Biofilm; Virulence	Huedo et al., 2014b, 2015
M30	Clinical (decubitus ulcer)	2	*cis*-11-Methyl-2-dodecenoic acid (DSF)	Virulence	Huedo et al., 2014b, 2015
R551-3	Environmental (endophyte of *Populus trichocarpa*)	1	*cis*-11-Methyl-2-dodecenoic acid (DSF)	Promote seed germination and plant growth	Alavi et al., 2013

NA*, Genomic data is not available.

As reported for the DSF synthases of *B. cenocepacia* (Bi et al., 2012) and *Xcc* (Zhou et al., 2015), both the RpfF-1 and RpfF-2 proteins from *S. maltophilia* appear to have a double acyl-ACP dehydratase and thioesterase activity that catalyze the conversion of (*R*)-3-hydroxy-11-methyl-dodecanoyl-ACP to DSF in two steps (Huedo et al., 2015). In addition, the thioesterase activity of all RpfF proteins seems to be nonspecific, cleaving a variety of medium- and long-chain acyl-ACP substrates and thus generating free FAs that are then released to the extracellular environment (Bi et al., 2012; Huedo et al., 2015; Zhou et al., 2015). In *S. maltophilia* the major free FA released by this thioesterase activity is the 13-methyltetradecanoic acid (*iso*-15:0), which is also the most abundant FA in the phospholipids of both *Xanthomonas* sp. (Vauterin et al., 1996) and *S. maltophilia* (Kim et al., 2010). Surprisingly, *iso*-15:0 is actually considered a biomarker phospholipid FA for the Gram-positive group (Kaur et al., 2005) and seems to be present only in Gram-negative bacteria displaying DSF communication. Interestingly, DSF and *iso*-15:0 are generated through the same biosynthetic pathway (Heath and Rock, 2002), which suggests a potential connection between DSF production and membrane synthesis (Huedo et al., 2015).

In line with this observations, the presence of *iso*-15:0 in the medium appears to modulate DSF production in *rpf*-1 strains, perhaps because the intact RpfC-1 sensor (10 TMR) is able to detect this FA, thus liberating free active RpfF-1 capable of subsequent DSF synthesis (Huedo et al., 2015). Several other environmental factors modulate DSF synthesis in *rpf*-1 strains. For example, while rich media and 28°C seem to be the optimal culture conditions to achieve high amounts of DSF in the supernatant (Huedo et al., 2015), iron restriction has been found to induce DSF production through the Fur system in strain K279a (García et al., 2015).

**Figure 1:**
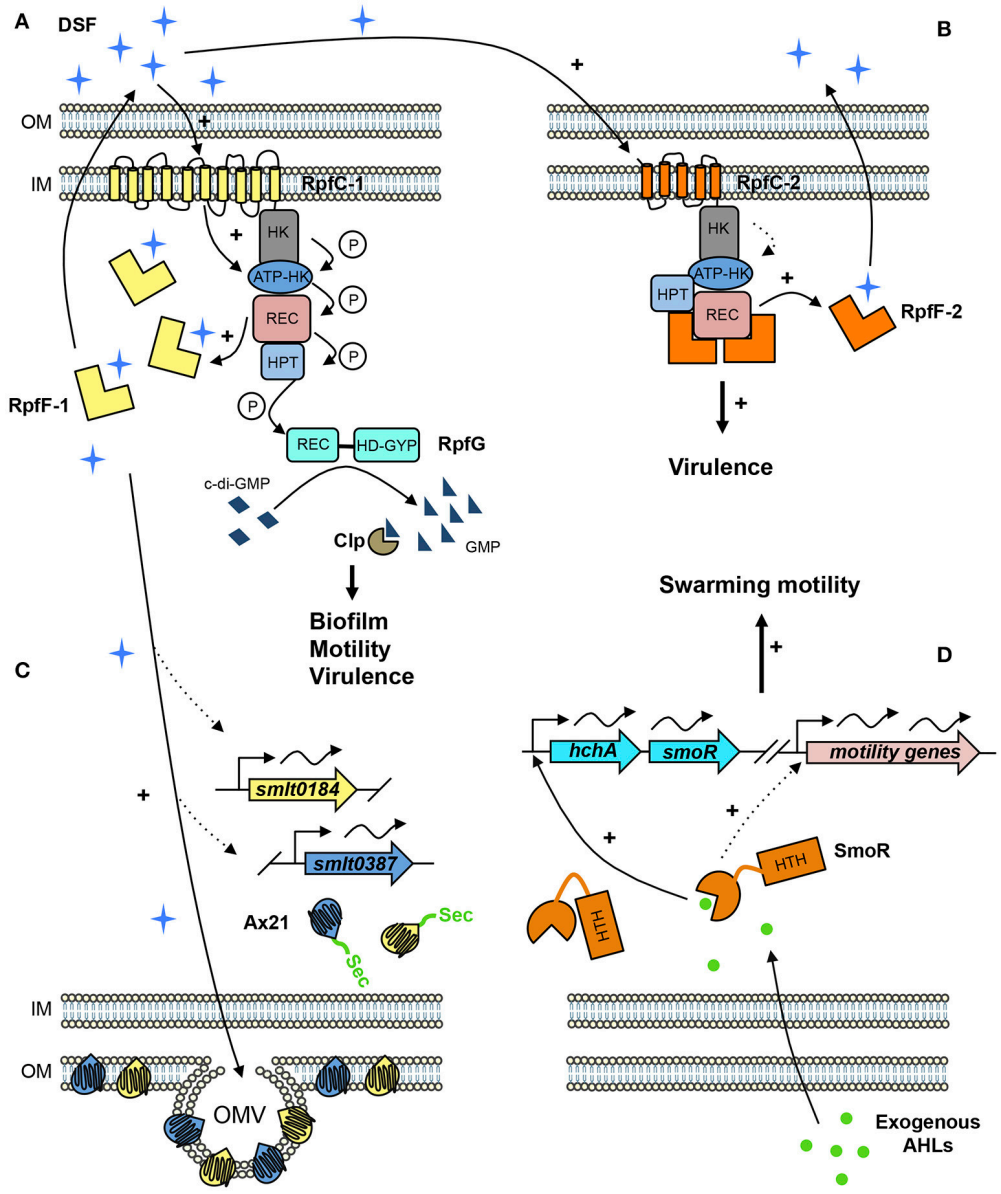
Proposed QS signaling network in *S. maltophilia*. **(A)** In *rpf*-1 strains, RpfC-1 (including 10 TMR) stimulates RpfF-1 basal activity—that increases with cell density—and synthesizes DSF (cis-11-Methyl-2-dodecenoic acid) that accumulates in the extracellular environment. Once DSF concentration reaches a critical threshold, RpfC-1 senses DSF, and induces a phosphorylation cascade throughout its cytoplasmic domains ending in the response regulator RpfG, which degrades cyclic diguanylate monophosphate (c-di-GMP) to GMP activating the transcriptional regulator Clp that stimulates expression of genes involved in biofilm formation, motility, and virulence. **(B)** In *rpf*-2 strains, RpfC-2 (5 TMR) permanently represses RpfF-2, resulting in no DSF detection in axenic conditions. DSF produced by neighboring bacteria (e.g., *rpf*-1 strain) is sensed by RpfC-2 allowing free-active RpfF-2 and subsequent DSF synthesis. **(C)** DSF also stimulates the production of outer membrane vesicles (OMV) containing high amounts of the two Ax21 proteins (Smlt0184 and Smlt0387). Both Ax21 proteins present a signal peptide that is processed by the general secretory (Sec) system. **(D)** Exogenous AHL signals, specifically C8-HSL and oxo-C8-HSL, are sensed by the LuxR solo SmoR (Smlt1839), annotated as “LuxR chaperone HchA-associated,” activating the transcription of its own operon and promoting swarming motility. Dotted lines represent predicted or supposed interactions on the basis of reported experimental evidences. Protein domains are abbreviated as follows. HK, Histidine kinase domain; REC, Receiver domain; HPT, Histidine phosphotransferase domain; HD-GYP, Phosphodiesterase domain containing an additional GYP motif; HTH, Helix-Turn-Helix domain.

Contrary to *rpf*-1 strains, DSF synthesis in strains harboring the *rpf*-2 allele seems to be permanently repressed under wild-type conditions. Nevertheless, the presence of exogenous DSF triggers DSF production in these strains (Huedo et al., 2015; Figure 1). These findings suggest that *rpf*-2 strains require a stoichiometric unbalance (RpfF-2>RpfC-2) for DSF production and that the 5-TMR sensor component of RpfC-2 is much more specific than RpfC-1, enabling free-active RpfF-2 only upon detection of DSF itself.

### Biological processes regulated by DSF in *rpf*-1 and *rpf*-2 strains

Deletion of *rpfF*-1 in the *S. maltophilia* clinical strain E77 resulted in altered biofilm formation, reduced bacterial motility and reduced virulence in the *Caenorhabditis elegans* and zebrafish models of infection (Huedo et al., 2014b). In the clinical model strain K279a (*rpfF*-1), interruption of the *rpfF* gene also resulted in decreased antibiotic resistance and protease secretion, and an altered lipopolysaccharide (LPS) (Fouhy et al., 2007). In the environmental strain WR-C, DSF-derivative signals stimulate flagella-independent motility (Huang and Lee Wong, 2007) and deletion of *rpfF* or *rpfB* decrease the expression of the ferric citrate receptor FecA (Huang and Wong, 2007). Recently, DSF produced by strain 44/33 has been shown to contribute to outer membrane vesicle (OMV) secretion (Devos et al., 2015; Table 1).

On the contrary, mutation of *rpfF*-2 does not significantly alter biofilm formation, bacterial motility or virulence in the clinical strain M30 (Table 1). This results are in line with the fact that the RpfF-2 variant seems to be permanently repressed (Huedo et al., 2014b). Nevertheless, when the *rpf*-1 and *rpf*-2 subpopulations cohabit, both DSF production and virulence capacity of the whole population are enhanced (Huedo et al., 2015; Figure 1). This suggests that *rpf*-2 strains have evolved as a receptor group, in terms of DSF communication, displaying a lethargic DSF-deficient phenotype under axenic conditions until the presence of DSF-producing bacteria (e.g., *Xcc* or *S. maltophilia rpf*-1 variant) triggers reciprocal DSF communication. This behavior evokes to some extend the *P. aeruginosa* “social cheaters”—spontaneous *lasR* mutants that take advantage of the intact QS-regulation of their neighboring bacteria (Sandoz et al., 2007). Clearly, further research is required to better understand the intriguing role of the DSF system in the *rpf*-2 *S. maltophilia* subpopulation, including the specific advantages and disadvantages of this particular behavior.

### DSF-mediated communication of *S. maltophilia* with distant organisms

*S. maltophilia* has been shown to interact, via DSF production, with unrelated bacteria, yeast, and even plants. In particular, DSF produced by *S. maltophilia* K279a is detected by *P. aeruginosa* through the sensor kinase PA1396, modulating biofilm formation and antibiotic resistance (Ryan et al., 2008) as well as virulence and persistence in lungs of cystic fibrosis patients (Twomey et al., 2012). Likewise, synthesis of DSF by the strain K279a affects planktonic and biofilm growth of *Candida albicans* and inhibits its morphological transition (de Rossi et al., 2014). Finally, DSF produced by the environmental strain R551-3 causes a positive effect on plant germination and growth of rapeseed (Alavi et al., 2013) (Table 1).

## AHL-based quorum sensing

N-acyl homoserine lactone (AHL) QS is the most studied and widespread communication system in Gram-negative bacteria (Papenfort and Bassler, 2016). Typically, AHL signals are produced by LuxI-type synthases and sensed by LuxR-type transcriptional regulators (Ng and Bassler, 2009; LaSarre and Federle, 2013).

### AHL synthesis in *Stenotrophomonas* species

It has been shown that *S. maltophilia* does not produce detectable levels of AHLs (Zhu et al., 2001; Veselova et al., 2003), reinforced by the lack of homologs to known AHL LuxI-family synthase genes in publicly available genomes. Nevertheless, AHL activity has been detected in some *Stenotrophomonas* sp. isolated from sediments of wastewater treatment systems (Valle et al., 2004; Hu et al., 2016) and activated sludge (Tan et al., 2014, 2015). Besides the *Stenotrophomonas* genus, AHL-activity has also been detected in other *Xanthomonadaceae* including *Thermomonas* (Ishizaki et al., 2017), *Lysobacter* (Tan et al., 2015) and *Xanthomonas* sp. (Veselova et al., 2003). Given the elevated genomic diversity of the genus *Stenotrophomonas*, future identification of more AHL-producing isolates or the existence of a novel LuxI-family synthase cannot be ruled out.

### AHL response in *S. maltophilia*

LuxR solos are typical AHL-regulators lacking its cognate LuxI and are widely spread throughout bacterial genomes, including *Xanthomonadaceae* species (Subramoni and Venturi, 2009; Hudaiberdiev et al., 2015). The genome of *S. maltophilia* strain K279 encodes for 15 putative LuxR solos from which only SmoR presents the typical N-terminal AHL-binding domain and the C-terminal helix-turn-helix (HTH) DNA-binding domain (Martínez et al., 2015). *In vitro* AHL-binding assays confirmed that SmoR from strain E77 binds to AHL signal oxo-C8-HSL, regulating swarming motility. The *S. maltophilia* E77 parental strain but not its derivative Δ*smoR* mutant strongly stimulates swarming motility in the presence of a *P. aeruginosa* supernatant (containing high levels of AHLs including oxo-C8-HSL), indicating that SmoR senses AHL signals produced by neighboring bacteria (Martínez et al., 2015) (Figure 1). The role of the other LuxR solos in *S. maltophilia* is yet to be elucidated.

## The proposed quorum-sensing factor Ax21

The small protein Ax21 (activator of XA21-mediated immunity in plants) was proposed to serve as a new QS mechanism in *Xanthomonadaceae* (Lee et al., 2009; Han et al., 2011; McCarthy et al., 2011; Ronald, 2011). However, after almost 10 years of research on the Ax21 protein, we are practically at the starting point, since the key studies proposing its function have been placed in doubt (Han et al., 2013; Lee et al., 2013; Bahar et al., 2014; McCarthy et al., 2017).

What appears to apply to *S. maltophilia*, based on two independent proteomic analyses, is that Ax21 is an outer membrane protein secreted in association with OMVs (Devos et al., 2015; Ferrer-Navarro et al., 2016). Interestingly, it has been found that the relative levels of the two Ax21 paralogs (K279a locus tags Smlt0184 and Smlt0387) in some *S. maltophilia* strains seem to correlate with their virulence potential (Ferrer-Navarro et al., 2013, 2016), and that the increase in OMV-associated secretion of Ax21 proteins is somehow regulated by the DSF-QS system (Devos et al., 2015) (Figure 1). Based on the evidences reported so far, we believe that Ax21 cannot be considered a QS system component itself. However, the link between DSF signaling, OMV production and Ax21 secretion, as well as the implication of this regulatory pathway on the virulence ability of *S. maltophilia*, should be further investigated.

## Quorum quenching involving *S. maltophilia*

The most studied QQ mechanisms are those disrupting AHL signaling (Wang et al., 2004), although QQ has been described for almost all QS systems including DSF (Newman et al., 2008; Defoirdt, 2017). Despite quenching of DSF-QS in *S. maltophilia* has not yet been reported, this species exhibits an interesting behavior in terms of QQ. It has been shown that the FA *cis*-9-octadecenoic acid synthesized by *S. maltophilia* strain BJ01 displays QQ of AHL signals resulting in antibiofilm activity on *P. aeruginosa* (Singh et al., 2013). AHL-QQ activity against 3-oxo-C12-HSL has been also observed in several *Stenotrophomonas* sp. and *S. maltophilia* isolates from activated sludge samples (Tan et al., 2015). Another study on activated sludge samples reported that one isolate from the genus *Stenotrophomonas* was able to degrade the C10-HSL signal (Ochiai et al., 2013). Endophytic isolates of *S. maltophilia* have been also shown to degrade 3-hydroxy palmitic acid methyl ester (3OH-PAME), the main QS signal of *Ralstonia solanacearum* (Achari and Ramesh, 2015). On the other side, detection, and response to AHL signals by *S. maltophilia* can be disrupted by the lactonase AiiA from *Bacillus subtilis* (Pan et al., 2008), resulting in non-swarming stimulation (Martínez et al., 2015).

Regarding the quenching of DSF-QS, *S. maltophilia* strain E77 grown in LB medium containing 5 μM of synthetic octadecanoic acid (18:0) reduces DSF production to undetectable levels (Huedo et al., 2015). Moreover, plant-associated bacterial species and particularly *Pseudomonas* spp. are able to rapidly degrade DSF molecules of *Xcc* (Newman et al., 2008), a mechanism that may apply against *S. maltophilia* DSF signals. Finally, DSF produced by *S. maltophilia* K279a inhibits the yeast-to-hyphal transition of *Candida albicans*, most probably by acting as antagonist of the *C. albicans* signal farnesoic acid, a DSF homolog (de Rossi et al., 2014).

In summary, *S. maltophilia* appears as a species with potential QQ applications. However, QQ mechanisms disrupting *S. maltophilia* signaling have never been reported.

## Concluding remarks and future perspectives

Research conducted during last years has significantly improved our understanding of cell-to-cell signaling processes in *S. maltophilia* but, at the same time, has aroused new questions and hypothesis.

The mechanistic processes of the DSF-QS system in the *rpf*-1 subpopulation seem highly similar to those reported for the DSF model organism *Xcc*. However, more efforts should be addressed to investigate the molecular basis of DSF-QS in the *rpf*-2 group (45% of isolates) in order to uncover the biological significance of this particular variant.

The sensing and quenching response of *S. maltophilia* to exogenous AHL signals suggests that this bacterium has evolved in close contact with AHL-producing bacteria. Given the high phenotypic and genotypic heterogeneity among isolates from the genus *Stenotrophomonas* and considering that AHL-producing isolates of *Stenotrophomonas* spp. have been already reported, the existence of *S. maltophilia* strains producing AHLs cannot be discarded and should be further investigated.

*S. maltophilia* clearly interacts with the organisms conforming its environment. Examples of cooperation via DSF are divers and include the stimulation of seed germination and growth of the rapeseed, but also an increment of biofilm formation and antibiotic resistance of *P. aeruginosa* in the lungs. However, in most known cases *S. maltophilia* appears to exert a negative effect on its competitors' QS systems. This is because *S. maltophilia* isolates possess an extraordinary array of QQ mechanisms including production of FAs with quenching activities as well as degradation of AHL and PAME signals.

Given the increasing incidence of multi-resistant isolates of *S. maltophilia* in clinical settings, new antimicrobial strategies should be explored. Exogenous mechanisms quenching DSF communication in *S. maltophilia* have not yet been investigated and may represent a promising approach to overcome bacterial multidrug resistance. With the knowledge on the DSF system increasing and particularly since the determination of the structure of the synthase RpfF and the sensor RpfC, designing and testing compounds with antagonist activity against these key QS components could provide further opportunities for the development of novel combination therapies with antibiotics.

Comprehensively, *S. maltophilia* appears to be extraordinarily well connected to its environment and to take part in inter-species communication by synthesizing sensing and degrading a wide range of signaling molecules, therefore actively participating in the decisions taken by the whole community.

## Author contributions

PH, XC, and DY conceptually designed the article and authored the first draft. XD, IG, and DY provided academic input and expertise, and finished critical revision of the article. All authors have approved the final version.

### Conflict of interest statement

The authors declare that the research was conducted in the absence of any commercial or financial relationships that could be construed as a potential conflict of interest.
